# Effects of fuzzless cottonseed phenotype on cottonseed nutrient composition in near isogenic cotton (*Gossypium hirsutum* L.) mutant lines under well-watered and water stress conditions^1^

**DOI:** 10.3389/fpls.2013.00516

**Published:** 2013-12-30

**Authors:** Nacer Bellaloui, Rickie B. Turley

**Affiliations:** Crop Genetics Research Unit, Plant Physiology, United States Department of Agriculture-Agricultural Research ServiceStoneville, MS, USA

**Keywords:** Boron, seed composition, cotton mutants, isogenic cotton, drought

## Abstract

There is no information available on the effect of fuzzless seed trait on cottonseed nutrient composition (minerals, N, S, protein, and oil) under drought stress. The objective of this research was to investigate the effect of the fuzzless seed trait on cottonseed nutrients using five sets of near-isogenic lines (NILs). Each set consists of two lines that share the same genetic background, but differ in seed fuzziness (fuzzy, F; fuzzless, N). The near isogenic lines will enable us to compare the effect of the trait without confounding the genotypic background effects. We hypothesized that since the fuzzless trait involved in fiber initiation development, and was reported to be involved in biochemical, molecular, and genetic processes, this trait may also alter cottonseed nutrient composition. Results showed that NIL sets accumulated different levels of minerals in seeds and leaves, and the fuzzless trait (N) in most of the lines altered seed and leaf mineral accumulations when compared with fuzzy lines (F) or the control line. For example, K, P, Mg, Cu, and Na concentrations in seeds were higher in MD N and STV N than in their equivalent MD F and STV F lines. Leaf concentrations of Ca, K, Mg, S, B, Cu, and Fe in MD N lines were higher than MD F line. Lower levels of nutrients in seeds and leaves were observed under water stress conditions, especially Ca, Mg, N, and B in seeds.Generally and with few exceptions, seed protein was higher in fuzzy lines than in fuzzless lines; however, seed oil was higher in fuzzless lines than in fuzzy lines. Our research demonstrated that fuzzless trait altered the composition and level of nutrients in seed and leaves in well watered and water stressed plants. Differences in protein and oil between fuzzy and fuzzless seeds may indicate alteration in nitrogen and carbon fixation and metabolism. The differential accumulation of seed nutrients in this germplasm could be used by cotton breeders to select for higher cottonseed quality.

## Introduction

Cotton is a major crop in the world, and it is important source for natural textile fiber, cottonseed oil and meal (Zhang et al., 2008), and minerals (He et al., 2013). Cottonseed contains about 17–27% oil and 12–32% protein (Kohel et al., 1985; Dowd et al., 2010; Yu et al., 2012). Cotton fiber yield and quality are influenced by biotic (diseases, genotype, and phenotype) and abiotic stress factors (drought, heat, mineral content in soil, and nutrients uptake and translocation) (Cherry et al., 1978; Kumar et al., 2012; Padmalatha et al., 2012). At least four cultivated cotton species are used, *Gossypium hirsutum* represents over 95% of the cultivated cotton, and *G. barbadense, G. arboretum*, and *G. herbaceum* together represent 5% worldwide (Padmalatha et al., 2012). Conventional cotton produces on its seed fuzz (hair on seed coat or fuzz fiber) and commercially valuable lint fiber. Development of mature cotton fiber has four stages: (1) initiation, i.e., expansion of epidermal cells and elongation of fiber initials (expansion of the cell to approximately 2–3 cm); (2) elongation of fiber initials; (3) secondary cell wall synthesis, which is characterized by the deposition of cellulose; and (4) maturation (drying of the fibers) (Naithani et al., 1982; Turley and Ferguson, 1996; Padmalatha et al., 2012). The final stage is maturation that takes place 45–50 dpa, and during this period fibers dehydrate and produce mature cotton lint (Ji et al., 2003; Arpat et al., 2004; Wilkins and Arpat, 2005). During the ginning process lint fibers are removed from the seed, but fuzz fiber is left behind because it is shorter. Two loci, N1 (dominant allele) and n2 (recessive allele), and either N- or n2n2 inhibit fuzz fiber development and produce “naked seed (Turley and Kloth, 2002).

Although the lint yield of fuzzless seed cotton is poor compared with conventional cotton (Turley and Kloth, 2002), the fuzzless trait has been used as a useful tool to gain knowledge of fiber initiation and development in the area of cell biology, genetics, and molecular biology. Recently, it was reported that transcriptome analysis showed major down regulation (90%) of transcripts at fiber initiation and early elongation (5 dpa) in the fuzzless mutant when *G. hirsutum* L. cv. MCU5 wild-type (WT) and its near isogenic fuzzless-lintless mutant where compared (Padmalatha et al., 2012). It must be noted that this line is different from our lines in that they initiate trichomes but the trichomes stop elongating very early. So far, no fuzzless seed alleles were reported. Padmalatha et al. (2012) observed down regulated transcripts at fiber initiation stage in the fuzzless mutant in Ca and phytohormone mediated signal transduction pathways, biosynthesis of auxin and ethylene and stress responsive transcription factors, transcripts involved in carbohydrate and lipid metabolisms, mitochondrial electron transport system, and cell wall loosening and elongation at fiber elongation stage (5–15 dpa). Down-regulated was also observed in the mutant cotton at fiber elongation stage (5–15 dpa), and cellulose synthases and sucrose synthase secondary cell wall biosynthesis stage (15–20 dpa). The following physiological and biochemical processes were also involved in fiber development: phytohormones such as ethylene (Shi et al., 2006), auxins (Yang et al., 2006; Zhang et al., 2011) and brassinosteroids (Sun et al., 2005; Luo et al., 2007); transcription factors such as MYB25 (Machado et al., 2009) and MYB25-like (Guan et al., 2011; Walford et al., 2011); osmotically active solutes such as soluble sugars, potassium and malate, ion-transporters such as H^+^-ATPases and K^+^-transporter that is important in maintaining the osmotic potential of the elongating fiber cell (Wang and Ruan, 2010); closure of plasmodesmata and the coordinated up-regulation of K+ and sugar transporters during fiber elongation to maintain the turgor pressure for the fiber cell elongation and the duration of plasmodesmata closure (Ruan et al., 2004); carbohydrate and energy metabolisms and their role in providing the carbon skeletons for cell wall polysaccharides and fatty acids synthesis (Gou et al., 2007; Pang et al., 2008, 2010a,b; Yang et al., 2008); the involvement of xyloglucan and pectin enzymes (Lee et al., 2010), arabinogalactans (Liu et al., 2008), and expansins (Harmer et al., 2002) in cell wall loosening and expansion during fiber elongation; secondary cell wall involvement during fiber elongation (Li et al., 2002, 2005; Wang et al., 2010); reactive oxygen species (ROS) homeostasis and its involvement in initiation and differentiation (Liu et al., 2012).

Nutrient involvement in the physiological and biochemical processes has been previously reported (Mengel and Kirkby, 1982; Marschner, 1995). For example, the role of calcium in promoting directional pollen germination and tube growth (Ge et al., 2009) and ovule fertilization (Faure et al., 1994; Digonnet et al., 1997) was investigated, and it was found that under heat stress conditions, ROS accumulated in plant tissues (Tang et al., 2006). This accumulation was accompanied by an increase in the cytosolic Ca levels of vegetative tissues (Gong et al., 1998; Jiang and Huang, 2001). This indicates that calcium is essential in enhancing antioxidant enzyme activity to protect the plant under oxidative stress conditions (Gong et al., 1998; Jiang and Huang, 2001). Exogenous Ca application has been shown to enhance antioxidant protection in heat-stressed leaves (Jiang and Huang, 2001). On the other hand, in elevated cytoplasmic Ca and in the presence of antioxidant enzymes, nicotinamide adenine dinucleotide phosphate oxidase produces O^−^_2_ to soften cell walls and promote cell expansion during pollen tube growth (Potocky et al., 2007). The indirect effect of nutrients on seed composition (protein and oil) is still not yet clear. Previous research indicated that maintaining optimum levels of nutrients such as N, C, K, B, and Zn in plant tissues and seeds is critical and has a significant influence on seed protein and oil quality (Bellaloui et al., 2009a,b). Also, it was reported that foliar nutrient application such as B resulted in higher seed protein, oleic fatty acid, and sugars, especially glucose, fructose, and sucrose (Bellaloui et al., 2009c, 2013). Sinclair and DeWitt (1975) and Shibles and Sundberg (1998) found that seed protein accumulation required a higher mobilization of stored N and S (Anderson and Fitzgerald, 2001) from vegetative tissues to seed, and seed N concentration is correlated with N availability within plants. It is clear that minerals and N and S play a major role in seed quality, and any alteration related to physiological, biochemical, or genetic processes can impact minerals, protein, and oil composition, affecting seed quality.

Nutrient uptake in cotton is controlled by nutrient supply and transport, and the absorption of the nutrients with water occurs due to a passive process (due to transpiration stream), protein transporters, concentrated nutrients in the xylem sap, or due to an active uptake process (requiring metabolic energy) (Bassirirad, 2000; Rochester et al., 2012). Nutrient uptake is driven by the demand for nutrients from the developing plant organs, and regulated by the supply of nutrients from the soil. Generally, the rate of nutrient uptake increases at flowering through fruiting, and then declines as the bolls mature (Mullins and Burmester, 2010). Because nutrient mobility within plants (phloem) differ between highly mobile (K, Mg, P, S, N, Cl, Na), intermediate (Fe, Zn, Cu, B, Mo) to low (Ca, Mn) ions (Marschner, 1995), N, P, K, Fe, Cu and Zn levels normally decline in leaf tissue as the plant ages, whereas Ca, Mg, Na, Mn, S and B increase in luxury supply). Declines in the leaf concentrations of N, P, K, Fe, Cu, and Zn may indicate redistribution of nutrients from foliage to the developing bolls. During flowering, critical leaf K concentrations which impact plant growth, physiology and yield ranged from 0.6 to 2.45% (Hsu et al., 1978; Oosterhuis et al., 2003), although narrower ranges have been also reported (Reddy and Zhao, 2005). Since the mature lint contains 80–95% cellulose, nutrients do not accumulate at high levels in lint (Meinert and Delmer, 1977; Beasley, 1979). The boll wall contains between 32 and 60% of K in the boll (Bassett et al., 1970; Kerby and Adams, 1985; Mullins and Burmester, 2010), and more than 70% of K and Ca remains in the boll walls and do not move to the seed. However, over 60% of N, P, Mg, Fe, Cu and Zn in bolls are translocated to seed, while Mn and B remain in boll walls. Role of K in cotton was reported to maintain cotton fiber cell turgor pressure, facilitating cell growth and secondary cell wall deposition (third phase of fiber initiation). Also, nutrients such as N, P, and Zn, taken up by the crop, were found to be redistributed into the developing bolls and removed in seed cotton (Rochester, 2010).

Since there is no information available on the effects of the trait “fuzzless seed” on nutrient composition in leaves and seed, the objective of this research was to study the effects of fuzzless cottonseed phenotype on cottonseed nutrient composition (minerals, N, S, protein and oil) in near isogenic cotton lines mutant for fuzzless seed trait. Since the Midsouth USA region, where these lines were developed, is subjected to drought, the experiment was also conducted under water stress conditions.

## Materials and methods

A greenhouse experiment was conducted where one set of plants were grown in well watered conditions (W) and the other set was grown under water stress (WS). Each set (W or WS) was repeated twice. To induce WS, soil in pots were weighed, and then saturated with deionized water and left to drain and weighed again to obtain the water field capacity as measured by soil water sensors inserted in pots (Bellaloui et al., 2013). Soil water potential in well watered soil was kept between −15 and −20 kPa (this was considered field capacity for a Dundee silt loam, fine-silty soil) and was between −90 and −100 kPa in soil of WS ed plants. Soil water potential was monitored daily by sensors inserted in the pots and read using Soil Moisture Meter (WaterMark Company, Inc., Wisconsin, USA). The mutant lines and their equivalent wild type were: Sure Grow 747 (SG F and SG N); Mississippi Delta 51*ne* (MD F and MD N); Stoneville 7A *gl* (STV F and STV N); Delta Experiment Station 119 (DES F and DES N); and 243 [(source of the dominant allele (N1, N1) (PI 528610)]. Cotton seeds were germinated and uniform size seedlings were transplanted into 9.45 L size pots filled with field soil. The soil was a Dundee silt loam (fine-silty, mixed, active, thermic Typic Endoqualfs) with pH 6.3, 1.1% organic matter, a cation exchange capacity of 15 cmol/kg, and soil textural fractions of 26% sand, 56% silt, and 18% clay. The concentrations of nutrients in soil were (mg kg^−1^): P = 474, K = 1217, S = 41, Ca = 1721, Mg = 1258, Mn = 195, Na = 54, B = 1.4, Zn = 23, Fe = 6094. The percentage of N and C were 0.6 and 0.81, respectively. Greenhouse conditions were about 34 ± 11°C during the day and about 28 ±6°C at night with a photosynthetic photon flux density (PPFD) of about 800–2400 μmol·m^−2^·s^−1^, as measured by Quantum Meter (Spectrum Technology, Inc., Illinois, USA). The range of light intensity reflects a bright, sunny, or cloudy day. To avoid differences in the day-length between the two experiments, the two experiments were conducted simultaneously during the normal growing season (from April to October). To further investigate the effect of foliar K and B on K and B mobility from leaves to seeds at boll development, K_2_ SO_4_ was applied at a rate of 4.0 lb ha^−1^ (4.5 kg ha^−1^) for three applications, and B form of H_3_BO_3_ was applied at a rate of 2.0 lb B acre^−1^ (1.8 kg ha^−1^) for two applications under irrigated and non-irrigated conditions. Only lines MD, STV, and 234 lines were used for the foliar application experiments and only three replicates were used.

### Minerals, N, and S analyses in leaves and seeds

The fully expanded leaves at boll development stage were collected from each treatment and each line and were analyzed for macro- and micronutrients. Cotton seeds at maturity were collected, ginned, acid-delinted, and analyzed for macro and micronutrients. Leaf and seed samples were ground to pass through a 1-mm sieve using a Laboratory Mill 3600 (Perten, Springfield, IL). Leaf and seed macro- and micro-nutrients were analyzed by digesting 0.5 g of dried ground seed in HNO_3_ in a microwave digestion system. The concentrations of nutrients were determined using inductively coupled plasma spectrometry (ICP) (Bellaloui et al., 2011). Nitrogen and S were measured in a 0.25-g sample using elemental analyzer (LECO CNS-2000, LECO Corporation, MI) (Bellaloui et al., 2011). Concentrations of B, Fe, and P were determined as indicated in the following sections.

### Boron determination

Boron in leaves and seeds was determined in dried ground seed with the azomethine-H method (Lohse, 1982). Calcium carbonate powder was added to seed samples before ashing to prevent losses of volatile B compounds. Briefly, 1 g of a dried ground seed sample was ashed at 500°C for 8 h, and after ashing, samples were extracted with 20 mL of 2 M HCl at 90°C for 10 min. After filtration, the filtrates were transferred to plastic vials, and then 2 mL of the filtrate solution was added to 4 mL of buffer solution (containing 25% ammonium acetate, 1.5% EDTA, and 12.5% acetic acid). A volume of 4 mL of azomethine-H solution containing 0.45% azomethine-H and 1% of ascorbic acid were freshly prepared before the analysis (John et al., 1975).

The concentration of B was determined after 45 min for color development using a Beckman Coulter DU 800 spectrophotometer (Fullerton, CA) at 420 nm.

### Cell wall B determination

Boron in cell wall was determined in leaves according to Hu and Brown (1994). Briefly, the fully expanding leaves were collected from each replicate and treatment and homogenized in mortar and pestle with ice in cold water. The resulting homogenate was spun in a centrifuge at 1000 g for 10 minutes, and the residue was washed three times with 10 ml of 80% ethanol and once with10 ml of methanol:chloroform mixture (1:1, v/v). Then, the precipitate was washed with 10ml of acetone, and samples were dried and ashed for cell wall B determination as described above in “Boron Determination” section.

### Iron determination

Leaf and seed iron concentration was measured after acid wet digestion, extraction, and reaction of the reduced ferrous Fe with 1,10-phenanthroline (Bandemer and Schaible, 1944; Loeppert and Inskeep, 1996). Briefly, 2 g of dried ground seed was acid digested (Analytical Methods Committee, 1959), then the acid was removed by volatilization and the soluble constituents were dissolved in 2 M of HCl. A solution of 0.25% (w/v) phenanthroline was prepared in 25% (v/v) ethanol, and the quinol solution (1% w/v) reagent was prepared on the day of use. A volume of 4 mL of the sample solution was added to a 25-mL volumetric flask, and the aliquot was diluted to 5 mL using 0.4 M HCl. Quinol solution (1 mL) was added and mixed. Then, 3 mL of the phenanthroline solution and 5 mL of the tri-sodium citrate solution (8% w/v) were added. The solution was diluted to 25 mL with distilled water and incubated at room temperature for 4 h. Iron concentration standard solutions were prepared in 0.4 M HCl, and ranged from 0.0 to 4 μ g mL^−1^ of Fe. The concentration of Fe was determined by reading the samples at 510 nm using a Beckman Coulter DU 800 spectrophotometer.

### Phosphorus determination

Phosphorus concentration in leaves and seeds was measured spectrophotometrically as the yellow phosphor-vanado-molybdate complex according to Cavell (1955) and Bellaloui et al. (2011). Briefly, a dried, ground sample of 2 g was ashed to completely destroy organic matter, and after ashing, 10 mL of 6 M HCl was added. The sample, then, was placed in a water bath to evaporate the solution to dryness. After drying, the sample was then put under heat and 2 mL of 36% v/v HCl was added and the sample was boiled. A volume of 10 mL of distilled water was added, and the solution was boiled, transferred to a 50-mL volumetric flask, diluted to 50 mL with distilled water, and filtered. A volume of 5 mL of 5 M HCl and 5 mL of ammonium molybdate–ammonium metavanadate reagent were added to 5 mL of the filtrate, and the solution was diluted with distilled water to 50 mL. The diluted solution was allowed to stand for 30 minutes before P measurement. Ammonium molybdate–ammonium metavanadate was prepared by dissolving 25 g of ammonium molybdate and 1.25 g of ammonium metavanadate in 500 mL of distilled water. The concentration of P was measured using a Beckman Coulter DU 800 spectrophotometer at 400 nm. Phosphorus standard solutions were prepared and the concentration of P, ranged from 0 to 50 μ g mL^−1^ of P, was made using dihydrogen orthophosphates.

### Cottonseed protein and oil

Mature cotton were collected from each treatment and each replicate and analyzed for protein and oil. Approximately 25 g of seed were ground using a Laboratory Mill 3600 (Perten, Springfield, IL). Analyses were conducted by near infrared reflectance according to Wilcox and Shibles (2001) and Bellaloui et al. (2009c) using a diode array feed analyzer AD 7200 (Perten, Springfield, IL). Calibrations were developed using Perten's Thermo Galactic Grams PLS IQ software. The calibration curve was established according to AOAC methods (1990a,b). Analyses of protein and oil were performed based on a seed dry matter basis (Wilcox and Shibles, 2001; Boydak et al., 2002).

### Experimental design and analysis

The experiment was arranged in a split plot design with irrigation as a main block and cotton lines as sub-plot. Four replicates were used for each treatment and for each sampling. Each pot with three plants was considered one replicate. Analysis of variance was conducted using Proc Mixed model in SAS (SAS, 2001). Means were separated by Fisher's least significant difference test at the 5% level of significance using SAS (SAS, 2001).

## Results

Analysis of variance showed that main effects of variety and watering treatments and their interaction were significant (*P* ≤ 0.0004) for K, Mg, P, N, B, Cu, Fe, Mn, Mo, Na, and Zn, indicating the significance of these factors as sources of variability, resulting in differential accumulation of nutrient concentrations in cottonseed (Tables 1, 2). Similar observation was recorded for leaf nutrients concentrations (Tables 3, 4). Since variety and watering treatments and their interactions were the main factors, and the patterns of nutrients in both experiments were similar, the data from the two experiments were pooled and combined. Therefore, each value in tables or figures represents eight replicates.

**Table 1:** **Analysis of variance (*F* and *P* values) of the effects of water treatment (Treat) on cottonseed macronutrients in fuzzless and fuzzy seed cotton isogenic mutant lines (Line) in greenhouse experiments (Exp)**.

**Source effect**	**DF**	**Ca**		**K**		**Mg**		**P**		**N**		**S**	
		***F***	***P***	***F***	***P***	***F***	***P***	***F***	***P***	***F***	***P***	***F***	***P***
Exp	1	0.04	0.9	5.1	0.0261	2.4	0.24	2.8	0.095	13.7	0.0003	7.8	0.006
Line	10	2.5	0.0098	9.1	<0.0001	12.3	<0.0001	19.0	<0.0001	6.7	<0.0001	1.4	0.209
Treat	1	1.0	0.31	118	<0.0001	147	<0.0001	1977	<0.0001	205	<0.0001	8.8	0.0035
Exp × Line	10	1.0	0.43	3.2	0.001	2.8	0.0031	1.7	0.099	0.9	0.5738	0.9	0.5647
Exp × Treat	1	4.2	0.05	0.4	0.51	2.6	0.11	7.9	0.006	10.3	0.0017	1.7	0.20
Line × Treat	10	0.5	0.87	4.4	<0.0001	4.8	<0.0001	3.5	0.0004	1.7	0.083	1.0	0.45

**Table 2:** **Analysis of variance (*F* and *P* values) of the effects of water treatment (Treat) on cottonseed micronutrients in fuzzless and fuzzy seed cotton isogenic mutant lines (Line) in greenhouse experiments (Exp)**.

**Source effect**	**B**		**Cu**		**Fe**		**Mn**		**Mo**		**Na**		**Zn**	
	***F***	***P***	***F***	***P***	***F***	***P***	***F***	***P***	***F***	***P***	***F***	***P***	***F***	***P***
Exp	4.4	0.28	0.03	0.85	15.4	0.0001	6.3	0.042	26.5	<0.0001	10.4	0.0016	1.3	0.31
Line	3.5	0.0004	26.8	<0.0001	9.8	<0.0001	29.2	<0.0001	10.6	<0.0001	21.0	<0.0001	17.9	<0.0001
Treat	54.8	<0.0001	228	<0.0001	129	<0.0001	457	<0.0001	230	<0.0001	385	<0.0001	314	<0.0001
Exp × Line	1.6	0.13	0.9	0.53	2.1	0.026	5.6	<0.0001	1.8	0.063	1.6	0.107	6.3	<0.0001
Exp × Treat	12.4	0.0006	1.8	0.19	20.9	<0.0001	21.8	<0.0001	4.5	0.036	18.4	<0.0001	3.1	0.079
Line × Treat	2.4	0.012	6.7	<0.0001	3.8	0.0001	14.7	<0.0001	3.4	0.0005	4.7	<0.0001	2.8	0.003

**Table 3:** **Analysis of variance (*F* and *P* values) of the effects of water treatment (Treat) on macronutrients in the fully expanded leaves at boll stage development in fuzzless and fuzzy seed cotton isogenic mutant lines (Line) in greenhouse experiments (Exp)**.

**Effect**	**DF**	**Ca**		**K**		**Mg**		**P**		**N**		**S**	
		***F***	***P***	***F***	***P***	***F***	***P***	***F***	***P***	***F***	***P***	***F***	***P***
Exp	1	0.02	0.8922	17.8	<0.0001	9.7	0.002	0.4	0.54	27.8	<0.0001	0.6	0.57
Line	10	15.7	<0.0001	19.5	<0.0001	15.6	<0.0001	33.6	<0.0001	3.7	0.0002	30.4	<0.0001
Treat	1	530	<0.0001	589	<0.0001	272	<0.0001	1481	<0.0001	762	<0.0001	366	<0.0001
Exp × Line	10	1.4	0.20	2.5	0.009	1.6	0.11	2.5	0.0096	2.8	0.0039	4.3	<0.0001
Exp × Treat	1	26.7	<0.0001	9.6	0.002	9.5	0.003	19.3	<0.0001	4.8	0.030	14.6	<0.0002
Line × Treat	10	4.2	<0.0001	6.2	<0.0001	4.0	<0.0001	26.0	<0.0001	12.3	<0.0001	7.1	<0.0001

**Table 4:** **Analysis of variance (*F* and *P* values) of the effects of water treatment (Treat) on micronutrients in the fully expanded leaves at boll stage development in fuzzless and fuzzy seed cotton isogenic mutant lines (Line) in greenhouse experiments (Exp)**.

**Effect**	**B**		**Cu**		**Fe**		**Mn**		**Mo**		**Na**		**Zn**	
	***F***	***P***	***F***	***P***	***F***	***P***	***F***	***P***	***F***	***P***	***F***	***P***	***F***	***P***
Exp	23.8	<0.0001	2.9	0.34	11.2	0.0129	0.6	0.45	0.5	0.57	0.2	0.74	53.1	<0.0001
Line	19.8	<0.0001	15.0	<0.0001	15.8	<0.0001	15.1	<0.0001	4.6	<0.0001	16.0	<0.0001	8.8	<0.0001
Treat	802	<0.0001	265	<0.0001	1195	<0.0001	1420	<0.0001	500	<0.0001	8.3	0.0045	366	<0.0001
Exp×Line	1.3	0.25	5.5	<0.0001	4.7	<0.0001	1.2	0.27	1.1	0.35	2.5	0.009	1.4	0.17
Exp×Treat	3.8	0.05	17.1	<0.0001	62.8	<0.0001	1.1	0.31	24.1	<0.0001	3.9	0.05	35.2	<0.0001
Line×Treat	18.2	<0.0001	14.0	<0.0001	13.2	<0.0001	13.3	<0.0001	3.5	0.0004	2.0	0.03	6.4	<0.0001

### Effect of fuzzless seed trait on seed nutrient composition under well-watered conditions

Concentrations of K, Mg, P, N, S, Cu, Fe, Mn, Mo, Na, and Zn were higher in fuzzless seed line STV N than in the fuzzy seed line STV F. Similar trend for nutrient concentrations was observed in SG, except for B, Fe, and Mn, and for MD except for Mo, B, and S (Table 5). The percentage increases of these nutrients in the fuzzless lines depended on line across isogenic sets and lines among each isogenic set. The range of seed nutrient concentration was also significantly different between lines in each set and lines between isogenic sets (Table 5).

**Table 5:** Characterization of cottonseed nutrients composition as influenced by fuzz (F) and fuzzless (N) seed trait in cotton isogenic mutant lines under well watered conditions^*^.

**Line**	**Ca (%)**	**K (%)**	**Mg (%)**	**P (%)**	**N (%)**	**S (%)**	**B (mg/kg)**	**Cu (mg/kg)**	**Fe (mg/kg)**	**Mn (mg/kg)**	**Mo (mg/kg)**	**Na (mg/kg)**	**Zn (mg/kg)**
243	0.1	1.2	0.3	0.7	3.9	0.3	14.8	18.3	68.9	16.7	2.3	305	64.7
56F	0.1	1.2	0.3	0.7	4.4	0.9	15.1	18.5	66.9	20.7	2.2	295	68.5
56N	0.3	1.0	0.3	0.7	4.7	0.4	15.3	20.2	65.7	19.1	2.0	343	71.0
DESF	0.3	0.9	0.4	0.7	4.7	0.3	15.2	18.9	65.6	15.6	2.0	283	63.9
DESN	0.2	1.2	0.4	0.8	4.5	0.5	15.1	12.8	94.7	22.7	2.5	377	71.2
MDF	0.1	1.0	0.3	0.6	3.8	0.3	15.6	21.9	62.8	17.6	2.4	200	56.8
MDN	0.1	1.2	0.4	0.8	5.1	0.4	15.5	36.3	89.8	28.6	2.4	449	89.5
SGF	0.1	1.1	0.4	0.7	4.0	0.4	16.5	28.8	74.1	17.5	2.7	383	86.8
SGN	0.1	1.3	0.5	0.9	4.1	0.4	18.6	45.0	78.0	17.8	3.2	376	71.2
STVF	0.1	1.1	0.3	0.7	3.7	0.3	16.1	25.0	65.1	17.6	2.4	316	87.2
STVN	0.1	1.3	0.5	0.9	4.9	0.4	16.3	39.4	96.6	23.8	3.7	446	92.5
LSD	0.04	0.05	0.02	0.02	0.22	0.16	0.70	2.5	5.6	1.4	0.18	23	4.1

* Values are means of eight replicates. The experiment was repeated twice.

### Effect of fuzzless seed trait on leaf nutrient composition under well watered conditions

Compared with the fuzzy line, the fuzzless line STV N showed higher concentration of leaf Ca, K, Mg, P, N, S, Fe, Mn, Mo, Na, and Zn, but not B and Cu. Fuzzless line MD showed higher concentrations of Ca, K, Mg, P, S, Fe, Mn, and Na, but not in N, Cu, Mo, and Zn (Table 6). Compared with the fuzzy line, the fuzzless line MD exhibited higher concentration of Ca, K, Mg, P, Fe, Mn, Mo, Na, and Zn in leaves, but not N and S. The fuzzless line 56N showed either no changes in nutrient concentrations or opposite trend to that of lines STV, MD, SG, and DES. The concentration of nutrients in leaves in lines among and between isogenic sets varied widely. For example, the percentage (%) ranged from 2.6 to 4.6 for Ca, 1.2–3.9 for K, 0.7–1.2 for Mg, 0.6–1.3 for P, 2.9–4.1 for N, 0.7–2.3 for S; the concentration (mg kg^−1^) for B ranged from 185 to 251, Cu from 11.6 to 43.6, Fe from 82 to 140, Mn from 159 to 289, Mo from 2.4 to 4.2, Na from 3407 to 6122, and for Zn from 22.2 to 50.2 (Table 6). The percentage (%) increase of nutrients in leaves of fuzzless lines depended on the line across isogenic sets, and ranged from 35 to 45 for Ca, 4–68 for K, 14–71 for Mg, 40–100 for P, 53–150 for S, 36–44 for B, 30–71 for Fe, 47–62 for Mn, 41–62 for Na, and 35–115% for Zn.

**Table 6:** Characterization of leaf nutrients composition as influenced by fuzz (F) and fuzzless (N) seed trait in cotton isogenic mutant lines under well watered conditions^*^.

**Line**	**Ca (%)**	**K (%)**	**Mg (%)**	**P (%)**	**N (%)**	**S (%)**	**B (mg/kg)**	**Cu (mg/kg)**	**Fe (mg/kg)**	**Mn (mg/kg)**	**Mo (mg/kg)**	**Na (mg/kg)**	**Zn (mg/kg)**
243	3.6	3.0	0.7	0.6	4.1	0.7	185	24.9	108	159	3.8	5914	29.1
56F	4.1	3.2	1.0	0.7	3.9	1.6	202	43.6	127	211	3.2	6114	34.5
56N	3.5	3.2	0.7	0.7	3.5	1.4	184	31.0	114	279	2.9	6122	33.0
DESF	2.6	2.9	0.7	0.6	3.2	1.3	167	15.0	82	289	2.7	3876	23.4
DESN	3.8	3.3	0.8	1.2	3.2	1.0	241	23.3	140	286	3.3	5483	50.2
MDF	3.4	2.4	0.7	0.7	3.5	1.5	174	15.3	122	171	4.2	3407	30.6
MDN	4.6	3.9	1.1	1.3	3.3	2.3	236	11.6	159	251	3.0	5079	30.8
SGF	4.0	2.3	0.7	0.6	3.2	1.0	204	14.8	119	271	3.6	3684	30.3
SGN	3.7	3.2	0.7	0.6	3.5	1.0	182	14.6	139	194	3.8	3695	27.3
STVF	2.9	2.2	0.7	0.5	3.4	0.8	178	15.4	83	162	2.4	3692	22.2
STVN	4.2	3.7	1.2	0.7	2.9	2.0	251	14.3	138	263	3.1	5970	29.9
LSD	0.21	0.20	0.06	0.04	0.79	0.11	9	3.1	9	14	0.79	312	3.2

* Nutrients were determined in the fully expanded leaves at boll stage development.

Values are means of eight replicates. The experiment was repeated twice.

### Effect of fuzzless seed trait on seed nutrient composition under water stress conditions

Cottonseed in MD lines showed higher concentrations of K, Mg, N, B, Cu, Na, and Zn in fuzzless seed than in fuzzy seed (Table 7). Line STV showed higher concentrations of P, N, S, Cu, and Fe in fuzzless seed than in fuzzy seed. Seeds in DES accumulated higher N, B, Fe, Mn, Mo, and Na in their fuzzless seed than in fuzzy seed. In SG, the concentration of K, P, Cu, Mn, and Mo were higher in fuzzless seed than in fuzzy seed. In line 56, only B and Fe concentrations were higher in fuzzless seed than in fuzzy seed. Concentration of Ca was higher in fuzzy seed than in fuzzless seed in lines 56 and DES, opposing the general trend observed under well-watered conditions. In the rest of lines, there were no changes in Ca concentrations. Compared with the well-watered plants, the concentration of Ca in lines did not significantly differ from those grown under WS, except in line 56 and DES. The range of nutrient increase in fuzzless seed compared with fuzzy seed was less under WS than under well-watered conditions. The mineral concentrations in seed were significantly different among and between isogenic sets, and they ranged from 0.6 to 1.5% for K, 51 to 76.5 mg kg^−1^ for Fe, 7.8 to 22.6 mg kg^−1^ for Mn, 140 to 245 mg kg^−1^ for Na (Table 7). Percentage (%) increase of nutrient concentrations in fuzzless under WS was lower than under well watered conditions, and they ranged from 14 to 67 for K, 0–50 for Mg, 0–33 for P, 0–21 for N, 0–200 for S, 18–66 for Cu, 18–66 for Fe, 10–71 for Mn, 23–62 for Mo, 42–74 for Na, and 20–42 for Zn.

**Table 7:** Characterization of cottonseed nutrients composition as influenced by fuzz (F) and fuzzless (N) seed trait in cotton isogenic mutant lines under water stress conditions^*^.

**Line**	**Ca (%)**	**K (%)**	**Mg (%)**	**P (%)**	**N (%)**	**S (%)**	**B (mg/kg)**	**Cu (mg/kg)**	**Fe (mg/kg)**	**Mn (mg/kg)**	**Mo (mg/kg)**	**Na (mg/kg)**	**Zn (mg/kg)**
243	0.1	0.6	0.2	0.3	3.1	0.3	14.0	11.1	52.4	7.8	1.4	221	40.5
56F	0.2	0.8	0.3	0.3	3.1	0.3	13.2	11.2	56.9	9.2	1.6	212	49.6
56N	0.1	0.7	0.3	0.3	3.2	0.3	14.3	10.8	58.3	8.9	1.5	176	48.9
DESF	0.2	0.8	0.3	0.3	3.3	0.3	12.3	11.6	53.1	12.9	1.4	172	50.8
DESN	0.1	0.8	0.2	0.3	3.6	0.3	13.4	11.0	55.0	14.9	1.6	245	48.1
MDF	0.1	0.7	0.2	0.3	2.8	0.3	14.0	11.9	54.4	10.8	1.6	144	46.1
MDN	0.1	0.8	0.3	0.4	3.4	0.3	15.0	14.0	53.0	10.6	1.5	236	65.3
SGF	0.1	0.9	0.3	0.3	3.0	0.3	14.4	14.1	54.6	10.5	1.3	235	56.5
SGN	0.1	1.5	0.3	0.4	3.0	0.2	13.8	20.6	53.8	11.6	1.6	231	48.6
STVF	0.1	0.8	0.3	0.3	3.2	0.1	15.4	13.1	51.2	13.2	1.3	140	51.6
STVN	0.1	0.8	0.3	0.4	3.6	0.3	14.6	21.8	76.5	22.6	2.1	244	62.1
LSD	0.04	0.09	0.02	0.02	0.17	0.02	0.60	0.70	2.3	0.63	0.14	8	2.6

* Values are means of eight replicates. The experiment was repeated twice.

### Effect of fuzzless seed trait on leaves nutrient composition under water stress conditions

The concentration of Ca, K, Mg, P, N, S, Fe, Mg, Na in leaves of fuzzless cotton were higher than in fuzzy STV cotton (Table 8). Similar observation was noted in MD lines, except for P concentration where no P concentration differences was noticed between fuzzless and fuzzy lines. In DES lines, Ca, Cu, Mn, Na, and Zn concentrations were significantly higher in fuzzless than in fuzzy lines. For SG lines only K, N, and Fe were higher in fuzzless than in fuzzy lines (Table 8). Generally, the concentration of nutrients was lower in leaves under WS conditions compared with well watered conditions (Tables 6, 8). The range of nutrient concentrations differed among lines and between isogenic sets, and they had a wide range (Table 8). The concentration increase (%) of nutrients in leaves ranged widely. Cu and Zn concentrations did not change significantly.

**Table 8:** Characterization of leaf mineral composition as influenced by fuzz (F) and fuzzless (N) seed trait in cotton isogenic mutant lines under water stress conditions^*^.

**Line**	**Ca (%)**	**K (%)**	**Mg (%)**	**P (%)**	**N (%)**	**S (%)**	**B (mg/kg)**	**Cu (mg/kg)**	**Fe (mg/kg)**	**Mn (mg/kg)**	**Mo (mg/kg)**	**Na (mg/kg)**	**Zn (mg/kg)**
243	2.2	1.4	0.5	0.2	1.3	0.5	126	8.0	54.9	52.5	1.9	5386	16.4
56F	2.7	1.6	0.5	0.2	1.5	0.8	131	8.2	59.4	51.4	1.1	6234	16.8
56N	2.6	1.4	0.5	0.3	1.5	0.7	145	7.9	57.9	49.9	1.3	5317	16.3
DESF	1.3	1.3	0.3	0.2	1.5	0.6	148	7.6	56.1	44.8	1.3	4970	15.3
DESN	1.9	1.3	0.3	0.2	1.4	0.5	144	8.2	48.5	51.4	1.4	51.4	16.6
MDF	1.5	1.3	0.4	0.2	1.7	0.5	125	7.5	46.9	55.3	1.2	3926	15.9
MDN	2.8	2.4	0.6	0.2	2.2	0.9	148	7.6	60.4	87.8	1.2	4936	15.4
SGF	1.4	2.5	0.6	0.2	1.6	0.4	82	8.0	41.4	61.9	1.3	4711	15.9
SGN	1.6	2.5	0.4	0.2	1.8	0.4	68	7.8	47.5	55.9	1.3	4627	14.3
STVF	1.5	1.1	0.3	0.1	2.3	0.4	81	7.7	50.1	68.5	1.4	4627	14.4
STVN	1.7	1.3	0.7	0.3	2.8	0.9	85	7.9	64.8	110.0	1.3	6350	14.3
LSD	0.16	0.09	0.04	0.02	0.15	0.08	5	0.43	2.9	3.6	0.15	360	0.6

* Nutrients were determined in the fully expanded leaves at boll stage development.

Values are means of eight replicates. The experiment was repeated twice.

### Cottonseed protein and oil under well-watered and water stressed conditions

**Figure 1:**
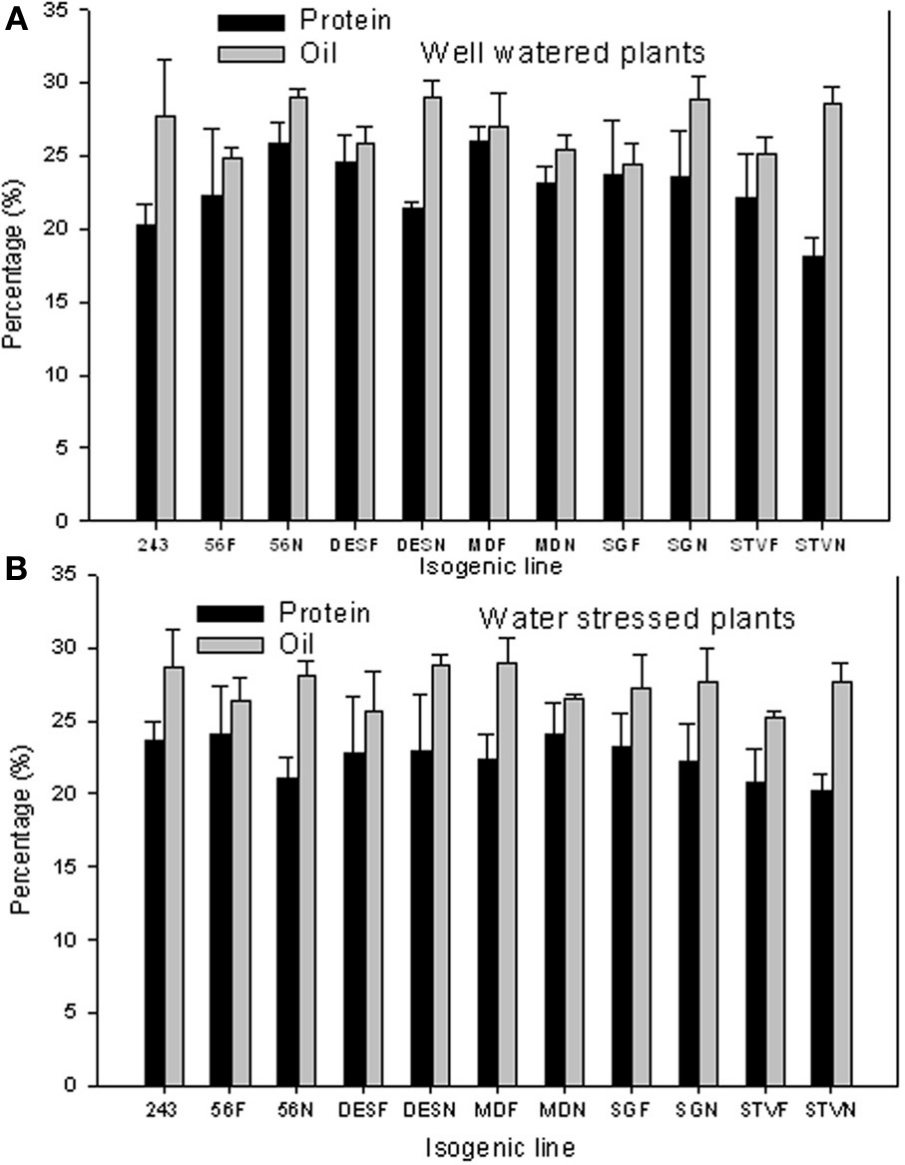
**Effect of fuzzless seed trait on seed protein and oil under well-watered (A) and water stressed (B) conditions in near isogenic cotton mutant lines**. In each line, F refers to a fuzzy line and N refers to its equivalent fuzzless line. Since the experiment was repeated twice and the results were pooled and combined, bar values are mean of eight replicates ± SE (standard error of the mean).

Protein and oil concentrations ranged widely between lines in each isogenic set and between isogenic lines across sets (Figures 1A,B). For example, under well watered conditions, the percentage differences between lines in each set reached 18% for protein and 17% for oil. Differences in protein between lines across isogenic lines reached 44% for protein and 18% for oil. Generally, and except for line 56, seed protein was higher in fuzzy seed lines that in fuzzless lines. However, seed oil was higher in fuzzless seed lines than in fuzzy lines, except in line MD. Under WS, the percentage differences between lines in each set reached 12% for protein and oil. Differences between lines across isogenic lines reached 20% for protein and 44% for oil. Under WS conditions, the trend of protein was similar to that under well-watered conditions for seed oil, but less clear for protein, except in line DES.

## Discussion

### Cottonseed and leaves nutrient composition under well watered conditions

Since the tested lines in each set differ only in the unique trait (fuzzless seed), the higher seed concentrations of K, Mg, P, N, Cu, Na, and Zn in fuzzless seed lines MD, SG, and STV can be attributed to the trait, resulting in differential accumulation of nutrients in seed. The higher accumulation of these nutrients in the fuzzless seed compared with the fuzzy seed may indicate that: (a) these nutrients were transported at higher rates during boll development, and this translocation could occur either directly as: i) a result of nutrient absorption by roots and transport through the xylem and then accumulation in bolls; (ii) or translocation of nutrients from leaves to bolls, or both. It must be noted that the translocation rate of nutrients differed, depending on the influx rate of each nutrient through roots and its mobility through the phloem; (b) nutrient requirement of fuzzless seed lines could be higher than the fuzzy seed lines, resulting in higher accumulation; (c) higher storage capacity for nutrients by fuzzless seed may be due to nutrients imbalance and signaling system and higher potential energy saving due to lack of fuzz on seed coat. Accumulation of nutrients in leaves in this experiment showed the same pattern as in seed. Nutrients such as B and Na showed unexpected higher concentrations in leaves, although there were no toxicity symptoms observed on plants during all stage of growth. For example, B concentration in leaves reached 251 mg kg^−1^ and Na 6122 mg kg^−1^. The concentration of some nutrient such as K, Ca, Na, and B in seed was much lower than in leaves, and this may be due to either limited translocation of these nutrients from leaves (source) to seeds (sink) or other unknown mechanisms such as alteration in hormones signaling system. Information available at the biochemical or physiological level explaining nutrients dynamic in fuzzless seed cotton is non-existent, and what is available is on cell biology, genetic, and molecular biology. For example, it was reported that there were changes in ovule proteins during early fiber development in a normal and a fibreless cotton (Turley and Ferguson, 1996) and reduction in β-glycerophosphatase and ATPase in postanthesis in epidermal cells of ovules from the fibreless line 9SO 3 HG (Joshi et al., 1985, 1988). Recent research on transcriptome analysis showed major down regulation of transcripts in the fuzzless mutant when MCU5 wild-type and its equivalent near isogenic fuzzless-lintless mutant where compared (Padmalatha et al., 2012). The down-regulation of energy metabolism, hormones signaling, and stress responsive processes observed in fuzzless mutants could result in nutrients imbalance signaling, leading to differences in nutrients mobility, transport, and nutrient accumulation between fuzzless and fuzzy isogenic lines during boll development. The differences in mineral concentrations can be supported by down-regulation of calcium and phytohormone mediated signals observed at fiber initiation stage in the fuzzless mutant (Padmalatha et al., 2012). It is clear that fibreless or fuzzless seed traits resulted in significant biochemical and molecular changes, which in turn may have impacted nutrients regulation. The only phenotype changes that are observed in these mutant lines used in the current study are fuzzless seed trait (the absence of fuzz fiber on the seed) and lower lint yields. The function of the fuzzless seed allele N1N1 appears to inhibit the initiation of cotton trichome development after one to two days after anthesis. By crossing a plant that is N1N1 (chromosome 12) with a plant that has the recessive fuzzless seed trait n2n2 (which is located on the homeologous chromosome 26 in the allotetraploid), a fibreless cottonseed with the genotype N1N1n2n2 was obtained. Although the real function is still unknown; however, RNA seq studies are planned for the near future. The NIL used in this study are theoretically about 98.46% pure by the standard backcrossing method used in breeding. Field studies on fiber quality and yields of these lines have just been completed, and more biochemical and cellular methods will be used to identify the gene responsible for this phenotype.”

Previous research in other species, using two sets of near-isogenic soybean lines for maturity genes, where each of the near-isogenic lines within a set had the same genotypic background, but differed in maturity genes, showed different accumulation of minerals in soybean seed, and this difference was due genotypic background and maturity genes for seed N, S, Ca, K, Mg, P, and B concentrations (Bellaloui et al., 2011). During this experiment, the contribution of maturity genes and genotypic background were quantified, and found to reach 43% for maturity and 84% for genotypic ground (Bellaloui et al., 2011). Research available in this area indicated down-regulation occurred in the mutant cotton at fiber elongation stage, cellulose synthases and sucrose synthase secondary cell wall biosynthesis stage, and minerals, especially K, Ca, and B. For example, the involvement of the following biochemical and physiological processes were reported during fiber development: osmotically active solutes such as soluble sugars, potassium and malate, ion-transporters such as H^+^-ATPases and K^+^-transporter and their involvement in maintaining the osmotic potential of the elongating fiber cell (Wang and Ruan, 2010); regulation of potassium (K+) and sugar transporters during fiber elongation to maintain the turgor pressure for the fiber cell elongation (Ruan et al., 2004); carbohydrate and energy metabolisms and their role in providing the carbon skeletons for cell wall polysaccharides and fatty acids synthesis (Gou et al., 2007; Pang et al., 2008, 2010a,b; Yang et al., 2008); involvement of pectin enzymes (Lee et al., 2010) and arabinogalactans (protein found in plant cell walls) (Liu et al., 2008) and expansins (non-enzymatic proteins found in the plant cell wall) (Harmer et al., 2002) in cell wall loosening and expansion during fiber elongation; and secondary cell wall involvement during fiber elongation (Li et al., 2002, 2005; Wang et al., 2010). In our experiment, and with few exceptions, cottonseed protein was higher in fuzzy seed than in fuzzless seed, but oil was higher in fuzzless seed lines than fuzzy seeds, possibly indicating energy saving differences and alteration in nitrogen and carbon metabolism. Further investigation to quantify carbon, carbohydrates, and fatty acids profiling in these lines should provide further understanding of physiological and biochemical changes due to the fuzzless seed trait.

### Cottonseed and leaf nutrient composition under water stress conditions

The lower concentration of nutrients in seeds and leaves in WS ed plants was due to lower soil moisture and lower uptake and limited translocation of nutrients from leaves to seed. The higher nutrient concentrations in leaves of fuzzless seed lines was noticed only for Ca, K, N, Na, and Fe, and not for others, indicating that the concentration and distribution of nutrients in leaves and seeds were influenced by both the fuzzless trait “gene” and its interactions with the environment, in our case WS. Since K and B were among the nutrients that showed higher concentrations in leaves and not in seed, a further investigation was conducted on K and B to observe the mobility dynamics and their distribution in leaves and seeds. This was achieved by conducting other experiment where K as K_2_SO_4_ was foliar applied at a rate of 4.0 lb ha^−1^ (4.5 kg ha^−1^) for three applications, and B as H_3_BO_3_ was foliar applied at a rate of 2.0 lb B acre^−1^ (1.8 kg ha^−1^) for two applications at bolls development under well-watered and water stressed conditions using only MD, STV, and 234 lines. Results (not shown) indicated that foliar K or B application increased K and B concentrations in leaves, bolls, and seed in all lines under well watered conditions. However, under WS conditions, foliar application of K and B resulted in higher concentrations of K and B in leaves and K in seed, but not significant increase of B in seed, and this may be due to higher mobility and translocation of K from leaves to seed during boll development. Also, translocation of B and K from leaves to seed was limited in the control fuzzless plants (no foliar K or B application) compared to K and B foliar applied plants. The limited translocation of K and B from leaves to seed in fuzzless seeds in MD, STV and SG, especially in WS conditions, is not understood, but could be due to nutrients regulation and hormones signaling imbalance, as suggested above. Further investigation on K and B mobility under different K and B supply is needed to understand nutrient partitioning in leaves and seeds.

### Cell wall boron vs. soluble boron in leaves of fuzzless and fuzzy seed lines under well-watered and water-stressed conditions

**Figure 2:**
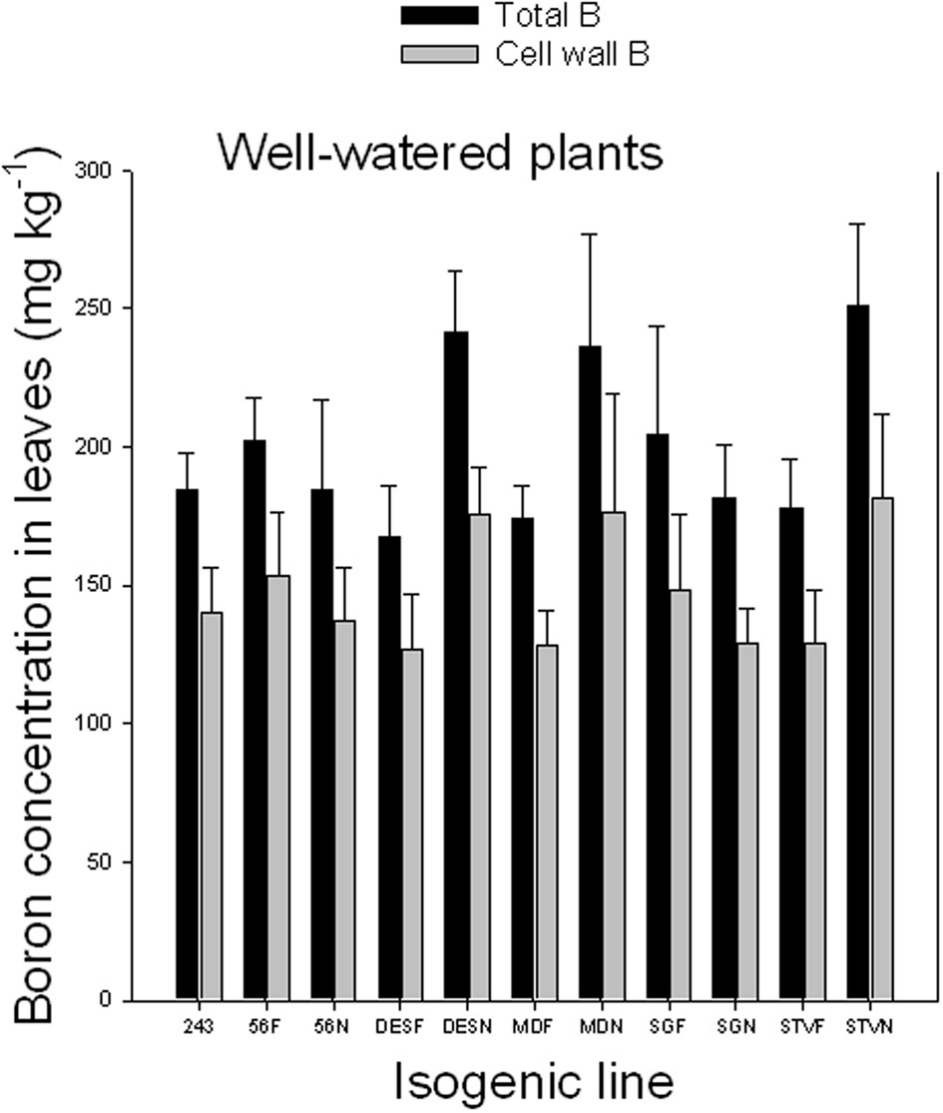
**Total and cell wall boron in leaves as affected by near isogenic mutant lines for fuzzless seed trait under well-watered conditions**. In each isogenic line, F refers to a fuzzy line and N refers to its equivalent fuzzless line. Since the experiment was repeated twice and the results were pooled and combined, bar values are mean of eight replicates ± SE (standard error of the mean).

**Figure 3:**
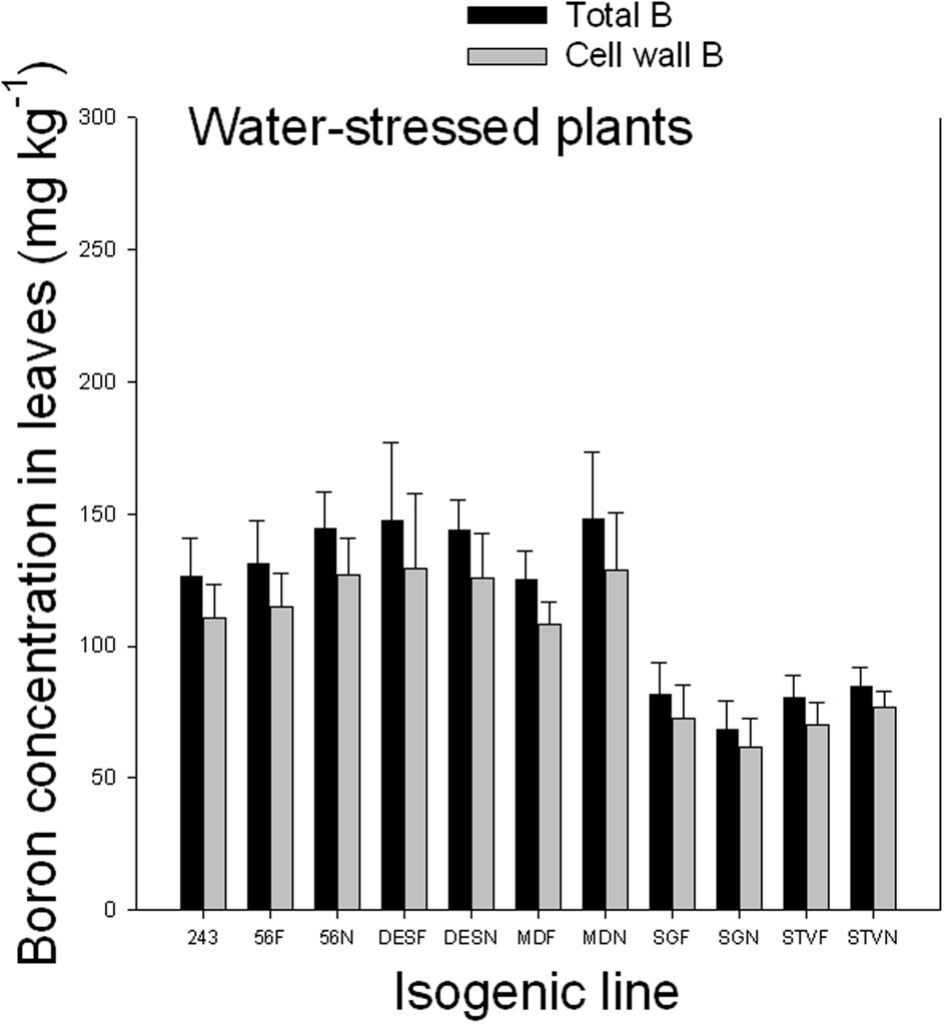
**Total and cell wall boron in leaves as affected by near isogenic mutant lines for fuzzless seed trait under water stressed conditions**. In each isogenic line, F refers to a fuzzy line and N refers to its equivalent fuzzless line. Since the experiment was repeated twice and the results were pooled and combined, bar values are mean of eight replicates ± SE (standard error of the mean).

Results indicated that cell wall B ranged from 71.4 to 75.9% under well watered conditions and from 86.6 to 90.5% under WS conditions (Figures 2, 3). Since the concentration of total B differed, depending on line among and between each isogenic sets, it appears that the isogenic lines have different B requirements, and the cell wall B is determined by the total B requirement, availability, and supply. The higher percentage of cell wall B, especially under WS conditions signifies the structural role of B in the cell walls, especially when B availability is limited. The structural role of B was previously reported and is well established. However, the exact metabolic function is not yet fully understood (Hansch and Mendel, 2009). It was reported that B is involved in the formation of meristematic tissues (Römheld and Marschner, 1991; Hansch and Mendel, 2009); stimulation or inhibition of enzymes, and phenol metabolism (Römheld and Marschner, 1991; Marschner, 1995); formation of borate esters with apiose residues of rhamnogalacturonan II (RG-II) in the cell wall (Kobayashi et al., 1996); contribution to the control of cell wall porosity and strength (Fleischer et al., 1999; Ryden et al., 2003); metabolism of carbohydtrates, RNA, and hormones such as indole acetic acid (Camacho-Cristóbal et al., 2008); induction of plasmalemma ATPase, increasing rate of transport of nutrients such as chlorine and phosphorus, and stimulating plasmalemma ATPase, leading to hyperpolarization of the membrane potential (Camacho-Cristóbal et al., 2008); absorption of ions and its deficiency may lead to lower uptake of several nutrients (Dugger, 1983); formation of reversible diester bonds with cis-diol-containing molecules and membrane stabilization crosslinking glycoproteins (Wimmer et al., 2009); and important for pollen germination and pollen tube growth (Agarwala et al., 1981). It was shown in cotton that B deficiency during flowering and fruit formation increases shedding, decreasing fiber yield and quality (Rosolem and Costa, 2000).

So far, there are three molecular mechanisms of B uptake and transport: (a) passive diffusion across lipid bilayers (Brown et al., 2002); (b) transport using major intrinsic protein (MIP) channels [boron permeation across root plasma-membrane vesicles (Dordas, 2006; Dordas et al., 2007)] or (AtNIP5;1), which belongs to the nodulin intrinsic proteins (NIP), subfamily of the MIPs family (Takano et al., 2006) and is localized and expressed in root epidermal, cortical, and endodermal cell plasma membrane, and found to be up-regulated in B-deficiency root (Takano et al., 2006); and (c) an energy-dependent high affinity transport system at low B supply mediated via BOR transporters (Dannel et al., 2002; Tanaka and Fujiwara, 2008). BOR transporters were previously identified, for example, OsBOR1 in rice and BOR1 involved and expressed under B deficiency for B efficient uptake into root cells (Takano et al., 2006; Nakagawa et al., 2007), or BOR transporter such as BOR4-GFP in *A. thaliana*, BOR1 and BOR2 for barley (Sutton et al., 2007) and barley and wheat (Reid, 2007), that were expressed at B toxicity level to increase B toxicity tolerance by pumping excess boric acid out of the cell (Miwa et al., 2007). The expression of these transporters under B deficiency or toxicity conditions can be applied to other species to develop crops tolerant to B deficiency or toxicity (Miwa and Fujiwara, 2010). The preferential transport of boric acid by non-sugar-alcohol-producing plants to young tissues under only B limitation, and not under normal B supply, suggests that plants can sense boron levels and regulate boron transport (Tanaka and Fujiwara, 2008), using a signaling system. Therefore, the mobility of B within the plant is dependent on plant species and B supply and availability. The agricultural implications of B mobility and re-mobilization is significant for B management, especially when B in soil is deficient or toxic (Rosolem and Bogiani, 2013).

## Conclusions

Our research showed that there is a wide range of nutrient concentrations in cottonseed in the introduced mutant germplasm. Our research demonstrated that lines of fuzzless seed had higher nutrient concentrations than fuzzy seeds, and this may be due to the fuzzless seed trait because the difference between two lines in each set was only the trait. This response may be due to: (a) physiological and biochemical alterations in nutrients demand and mobility within plants; (b) higher storage capacity for nutrients by fuzzless seed due to nutrients imbalance and hormonal signaling system; or (c) higher nutrient storage may indicate higher potential energy saving due to lack of fuzz on seed coat. The concentration of cell wall in lines across isogenic sets may be due to genotypic differences. The higher percentage of cell wall B, especially under WS conditions signifies the structural role of B in the cell walls under limited B availability conditions. The differences between fuzzless and fuzzy seed protein and oil may be due to alteration in nitrogen and carbon metabolism. The wide range of cottonseed protein and oil in this germplasm represents an opportunity for breeders to select for cottonseed with higher quality of protein and oil. To our knowledge, this is the first report on nutrients characterization in fuzzless and fuzzy cotton seed isogenic mutant lines. To investigate differences in energy sources, further research on carbohydrates and carbon dynamics and their partitioning in leaves and seeds of NILs is needed.

### Conflict of interest statement

The authors declare that the research was conducted in the absence of any commercial or financial relationships that could be construed as a potential conflict of interest.
